# Pasireotide protects mammalian cochlear hair cells from gentamicin ototoxicity by activating the PI3K–Akt pathway

**DOI:** 10.1038/s41419-019-1386-7

**Published:** 2019-02-06

**Authors:** Krystsina Kucharava, Marijana Sekulic-Jablanovic, Lukas Horvath, Daniel Bodmer, Vesna Petkovic

**Affiliations:** 1 Department of Biomedicine, University Hospital Basel, University of Basel, Basel, 4056 Switzerland; 2 Clinic for Otolaryngology, Head and Neck Surgery, University Hospital Basel, Basel, Switzerland

## Abstract

Gentamicin is a widely used antibiotic for the treatment of gram-negative bacterial infections; however, its use often results in significant and permanent hearing loss. Hearing loss resulting from hair cell (HC) degeneration affects millions of people worldwide, and one major cause is the loss of sensory HCs in the inner ear due to aminoglycoside exposure. Strategies to overcome the apparently irreversible loss of HCs in mammals are crucial for hearing protection. Here, we report that the somatostatin analog pasireotide protects mouse cochlear HCs from gentamicin damage using a well-established in vitro gentamicin-induced HC loss model and that the otoprotective effects of pasireotide are due to Akt up-regulation via the PI3K–Akt signal pathway activation. We demonstrate active caspase signal in organ of Corti (OC) explants exposed to gentamicin and show that pasireotide treatment activates survival genes, reduces caspase signal, and increases HC survival. The neuropeptide somatostatin and its selective analogs have provided neuroprotection by activating five somatostatin receptor (SSTR1–SSTR5) subtypes. Pasireotide has a high affinity for SSTR2 and SSTR5, and the addition of SSTR2- and SSTR5-specific antagonists leads to a loss of protection. The otoprotective effects of pasireotide were also observed in a gentamicin-injured animal model. In vivo studies have shown that 13 days of subcutaneous pasireotide application prevents gentamicin-induced HC death and permanent hearing loss in mice. Auditory brainstem response analysis confirmed the protective effect of pasireotide, and we found a significant threshold shift at all measured frequencies (4, 8, 16, 24, and 32 kHz). Together, these findings indicate that pasireotide is a novel otoprotective peptide acting via the PI3K–Akt pathway and may be of therapeutic value for HC protection from ototoxic insults.

## Introduction

Sensory hair cells (HCs) in the inner ear are the primary receptors of auditory signals^1^, and HC degeneration is the primary event in most cases of hearing loss. Hearing deficits can be caused by a variety of factors generating oxidative stress, including noise, infection, and ototoxic drugs such as aminoglycoside antibiotics and cisplatin. In mammals, auditory HCs are produced only during embryonic development^2^, and HC loss results in profound and irreversible deafness as there are currently no effective therapies.

Ototoxic drugs are the major environmental contributors to hearing loss. However, drugs including gentamicin, kanamycin, and tobramycin are used worldwide to combat serious gram-negative infections, in the treatment of tuberculosis, and in cystic fibrosis prophylaxis despite these side effects^3^.

Previous studies investigating gentamicin report its accumulation within the sensory HCs of the inner ear^4^, consistent with the gentamicin accumulation found in HCs across different animal species^5^. Gentamicin enters through the hair-cell transduction channel or is endocytosed via the apical surface^6,7^. HCs undergo apoptotic cell death when cellular protective mechanisms are overwhelmed by the toxic effects of free radicals. One strategy to maintain HC viability following ototoxic damage is to activate homeostatic and other protective mechanisms to promote hair-cell survival. Ototoxicity is the main dose-limiting factor in the clinical application of aminoglycoside antibiotics, and there is great interest in developing effective strategies to protect the inner ear without compromising anti-bacterial activity. Morphological evidence from many vertebrate species suggests that HC loss in response to aminoglycoside treatment occurs via apoptosis^8,9^.

We have focused on the neuroprotective actions of the neuropeptide somatostatin (SST) and its synthetic analogs octreotide and pasireotide, and have shown previously that SST and its analogs can protect mouse cochlea from gentamicin-induced HC loss in an in vitro system^10–12^. SST is a peptide hormone that exerts inhibitory effects by binding to specific cell-surface G protein-coupled receptors, of which five distinct subtypes have been characterized^13^. Binding of SST or its analogs to SST receptors (SSTRs) initiates a complex set of signaling events triggered by interactions between the activated receptors and a large number of different proteins, and involving the activation of specific G proteins. This is followed by the activation of phosphotyrosine phosphatases (PTPs), which directly interact with density-enhanced phosphatase 1 (DEP-1)/PTPη^14^. Pasireotide is a new cyclohexapeptide synthetic SST analog that binds to SSTR2 and SSTR5 with a high affinity. Pasireotide has a higher affinity to these receptors than either octreotide or lanreotide, and its affinity for SSTR5 is two times higher than that of SST itself. Pharmacokinetic studies have suggested that pasireotide does not undergo extensive hepatic metabolism and that it has a long terminal elimination half-life of 11 h^15^.

Studies have demonstrated that native SST inhibits secretion and induces proliferation, and induces several protective intracellular pathways, depending on the receptor subtype and target tissue^16^. Several key enzymes are involved, including PTPs^17^, adenylyl cyclase^18^, mitogen-activated protein kinase^13^, and phosphoinositol-3-kinase/Akt, which modulate Ca^+2^ influx^17,19^.

PI3K signaling is known to play an important role in HC development and has been implicated in the proliferation of otic progenitors, but its role, as a therapeutic target for promoting cell survival, has not been explored^18^. There is evidence to indicate that Akt is involved in the survival and protection of auditory  HCs in vitro^20–22^, AKT is the major downstream target of PI3K and PTEN, and acts as a phosphatase that modulates phosphotidyl inositol levels at the cell membrane to regulate PI3K signaling^23,24^.

Here, we report that pasireotide protects cochlear HCs against gentamicin ototoxicity in vivo and in vitro, functioning through the up-regulation of Akt. The Pi3K–Akt signaling pathway maintains HC integrity and functionality following otic insults such as gentamicin exposure.

## Materials and methods

### Animal care and handling

Experiments were performed on cochleae isolated from C57BL/6N mice (HARLAN Ltd., Füllinsdorf, Switzerland) of both sexes. All animals were maintained on a 12-h light/12-h dark schedule and had free access to water and standard mouse diet. One male and two female mice were placed in a single cage after 1 week of acclimation for breeding. Animals were maintained at the animal experimental station in ZLF (Hebelstrasse 20, 4030 Basel) at the Department of Biomedicine. The facility provides group housing, feeding with standard chow, and in-house breeding for colony maintenance. All animals were inspected regularly for health status, and the maintenance protocols were subject to the provisos of the animal welfare ordinance. Both adult and postnatal 5-day-old mice were used.

All animal procedures were conducted in compliance with the European Communities Council Directive of 24 November 1986 (86/609/EEC) and were approved by the Kantonales Veterinäramt, Basel, Switzerland.

### Organ of Corti tissue culture and drug treatment

Organs of Corti (OCs) were prepared as previously described^25^. Five-day-old (P5) C57BL/6N mice of both sexes were decapitated and cochlear microdissections were performed under a light microscope to isolate OCs. Brain tissue was contemporaneously removed. Isolated tissues were maintained in ice-cold phosphate-buffered saline (PBS) during microdissection, and OCs were subsequently incubated in culture medium (Dulbecco’s Modified Eagle's Medium) supplemented with 10% fetal calf serum, 25 mM 4-(2-hydroxyethyl)-1-piperazineethanesulfonic acid, and 30 U/ml penicillin (Invitrogen, Carlsbad, USA) at 37 °C and 5% CO_2_ for 24 h. Control OCs were incubated in culture medium only, without drugs. Hundred micromolar gentamicin was added after the 24-h incubation (Sigma-Aldrich, St. Louis, MO, USA) for an additional 24 h, and the drug remained present during gentamicin exposure. The gentamicin concentration (100 µM) reproducibly caused the loss of approximately 50% of HCs in a pilot titration. Five micromolar pasireotide (Novartis Pharma, Switzerland) and 10 µM SH-6 (Enzo Life Sciences, NY, USA) were added for 4 h as pretreatment.

Hundred micromolar CYN-154806-SSTR2 antagonist (Sigma-Aldrich, Switzerland) and 100 µM BIM 23056-SSTR5 antagonist (Sigma-Aldrich, Switzerland) were added separately or together for 1 h followed by an additional 4 h incubation with pasireotide. OCs were exposed to 100 µM gentamicin for 24 h after pretreatment, and the drugs remained present during the gentamicin phase.

To exclude the toxicity of above drugs, we incubated cell cultures in a medium solution containing the drugs at working concentrations for 24 h.

### Quantification of HCs

OCs were fixed in 4% paraformaldehyde in PBS and permeabilized by washing in PBS-T (0.1% Triton X-100 in PBS). Samples were then incubated with a 1:100 dilution of Texas Red X-phalloidin (Molecular Probes, Eugene, USA) in PBS-T for 40 min at 4 °C. The explants were mounted on a slide with Mowiol after several rinses with PBS. Phalloidin-stained stereociliary bundles and circumferential F-actin rings on the cuticular plate of the outer HCs (OHCs) and inner HCs (IHCs) allowed for the determination of present or missing cells. Only viable HCs were counted, with viability criteria including the presence of an intact cuticular plate with an intact stereociliary bundle. Cell populations were assessed on a fluorescence microscope (Olympus IX71) with images captured using an AxioCam system (Zeiss, San Diego, USA). The right objective had a 0.17-mm calibrated scale imposed on the field for reference, and the single row of IHCs and all three rows of OHCs were longitudinally oriented within each 0.17-mm frame. Each successive 0.17-mm field was evaluated for the absence of IHCs and OHCs beginning at the apex and moving down the OC to the base. Segments containing 60 OHCs associated with 20 IHCs in a given microscopic field were included in the quantitative analyses. IHCs and OHCs were counted and these values were used to calculate cell survival. The average number of OHCs and IHCs was determined for each explant by counting HCs in three segments selected randomly from the basal turn and three segments from the apical turn. Each group included 8–10 mice (*n* = 8–10; 16–20 OCs) with three experimental replicates.

### RNA isolation and quantitative PCR

OCs intended for RNA isolation were stored in RNAlater (Ambion, USA). RNA was isolated with a Direct-Zol RNA MiniPrep kit (Zymo Research, USA) according to the manufacturer’s instructions. The quantity and quality of isolated RNA were determined with a NanoDrop 1000 (ThermoScientific), and the 260/280-nm absorbance ratios were between 1.8 and 2.1 for all samples. Total RNA (1000 ng) was reverse transcribed into cDNA with High-Capacity cDNA Reverse Transcription Kit (Applied Biosystems, USA). Quantitative PCR (qPCR) was performed with an ABI Prism 7900HT Sequence Detection System (Applied Biosystems, USA) and with Power Sybr Green Master Mix (Applied Biosystems, USA). The primer sequences used for qPCR were (5′–3′) *Nfkb1* forward: ATG GCA GAT GAT CCC TAC, reverse: TGT TGA CAG TGG TAT TTC TGG TG; *Akt 1* forward: GCC AAA GTC CAG CAA GAA GG, reverse: CTG AAC CGC ATG GGA CAC AG; *Pi3K* forward: CGA GAG TGT CGT CAC AGT GTC, reverse: TGT TCG CTT CCA CAA ACA CAG; *Gapdh* forward: TGA CCT CAA CTA CAT GGT CTA CA, reverse: CTT CCC ATT CTC GGC CTT G; *Gab1* forward: 5′-GAA GTT GAA GCG TTA TGC GTG-3′, reverse: 5′-TCC AGG ACA TCC GGG TCT C-3′; *Ptpn7* forward: 5′-GGT TGA ACC CAT CTG CTC AGT-3′, reverse: 5′-CTG TAG CGT CCA GCG TGT AA-3′; and *Gpr1* forward: 5′-CAT CGG CTT ATG TGA AGC TGG-3′, reverse: 5′-CAG ATG GCA GAG CGT ATG C-3′ (Microsynth, St. Gallen, Switzerland). The final primer concentration was 250 nM per reaction. The thermocycling parameters were 10 min at 95 °C, then 40 cycles of 95 °C for 15 s, and 60 °C for 60 s. Template-free controls ensured that nonspecific amplification and DNA contamination could be excluded. The relative quantities of specifically amplified cDNAs were calculated with the comparative threshold cycle method, and Gapdh expression levels were used as the endogenous reference.

### Caspase assay

Apoptosis was determined using a Caspatag Pan-Caspase detection kit (Millipore/Chemicon, Germany). After proceeding with the caspase staining according to the manufacturer’s instructions, the OC explants from 5-day-old mice were fixed with 4% paraformaldehyde in PBS at room temperature (RT) for 20 min and permeabilized by washing in PBS-T. The samples were then incubated in a 1:100 dilution of rhodamine-phalloidin (Molecular Probes, Eugene, USA) in PBS-T for 40 min at 4 °C. The explants were mounted on a slide with Mowiol after several rinses with PBS. The OC was observed in whole mounts using a fluorescence microscope (Olympus IX71) and images captured by an AxioCam system (Zeiss, San Diego, USA) to measure the signal intensity of the defined regions of interest, which was the same for all OCs compared. Green fluorescence intensity was proportional to the amount of active caspase in this system. Images were processed and analyzed using Fiji-win 32 software, the intensity of the green signal was measured, and the background was subtracted. The defined region of interest was the same for all the images, and the brightness was calibrated in the range of 0–255 arbitrary units.

### Western blotting

Protein samples were prepared from the cochlea of 5-day-old pups. Experimental OCs were treated as described above and placed in cell lysis buffer with a protease inhibitor cocktail (Sigma; C3228, P8340), and then homogenized for 1 min on ice. Protein concentrations were measured with a NanoDrop 1000 (ThermoScientific), and mouse brain lysates were used as the control. After measurement, the lysates were mixed with an equal amount of Laemmli sample buffer (Sigma) and heated at 95 °C for 5 min. Samples (10 μg protein per lane) were resolved using sodium dodecyl sulfate-polyacrylamide gel electrophoresis. Proteins were blotted onto a polyvinylidenefluoride membrane after electrophoresis, and nonspecific sites were blocked with 5% non-fat dry milk dissolved in PBS for 1 h at RT. Membranes were then incubated with primary antibodies in PBS-Tween. The following antibodies were used: primary: rabbit polyclonal phospho-Akt 1:1000 (Cell Signaling, Switzerland), rabbit polyclonal Akt 1:1000 (Cell Signaling, Switzerland), rabbit polyclonal phospho-PTBη (subunit1B) 1:1000 (Abcam, UK), and mouse monoclonal PTBη (subunit1B) 1:500 (Abcam, UK) and secondary: HRP-linked anti-Rabbit secondary antibody 1:2000 and anti-mouse 1:3000 (Cell Signaling, Switzerland). After an overnight incubation with primary antibodies at 4 °C, membranes were washed with PBS-Tween (3 × 10 min) and incubated with an appropriate HRP-conjugated secondary antibody for 1 h at RT. After washing, bands were visualized with Super Signal West Dura Extended Duration Substrate (ThermoScientific, Switzerland). We used rabbit polyclonal β-actin 1:1000 (Cell Signaling, Switzerland) to demonstrate equivalent protein loading.

### Drug administration

A total of 36 (18 female and 18 male) C57BL/6N 5- to 7-week-old mice were randomly appointed to three groups: group 1 (control, *n* = 12), which received normal saline 0.1 ml intraperitoneally (i.p.) once a day for 10 days; group 2 (gentamicin, *n* = 12), which received a single injection of gentamicin (180 mg/kg) followed by furosemide (100 mg/kg) administered 40 min after gentamicin once a day for 10 days; and group 3 (gentamicin + pasireotide, *n* = 12), which received a single subcutaneous injection of pasireotide (50 mg/kg) once a day for 3 days as a pretreatment and 4 h prior to a single injection of gentamicin (180 mg/kg) in combination with furosemide (100 mg/kg) for 10 days.

All drugs were dissolved in sterile physiological saline according to mouse bodyweight. Animals received a single dose of gentamicin (180 mg/kg i.p.; Dr. E. Graeub AG, Bern, Switzerland) followed by a single dose of furosemide (100 mg/kg i.p.; MSD Animal Health GmbH, Luzern, Switzerland) 40 min later. A single dose of pasireotide (50 mg/kg i.c.; Novartis Pharma, Basel, Switzerland) was used for pretreatment and for the combination with gentamicin/furosemide.

### Hearing function tests

All animals received auditory brainstem response (ABR) tests using Tucker-Davis Technologies (TDT-RZ6-A-P1) hardware and software (Alachua, FL, USA) before treatment. Mice were anesthetized with a single i.p. injection of 80 mg/kg BW ketamine (Graeub AG, Switzerland), 12 mg/kg BW xylazine (Graeub AG, Switzerland), and 2 mg/kg acepromazin (Arovet AG, Switzerland). All ABRs were performed in sound-attenuating acoustic chambers, and the body temperature of the animals was maintained at 38 °C with an infrared warming pad (Kent Scientific Corporation, USA). The tone-burst acoustic stimuli were synthesized with BioSig software (10 ms duration; 0.5 ms rise/fall time; Blackman window), amplified using an SA1 audio amplifier, and transduced by a Multi Field Speaker-Stereo (MF1-S). A non-inverting needle electrode was placed at the midline vertex, the inverting electrode was placed over the mastoid of the tested ear, and the ground electrode was placed on the upper thigh. An electrostatic speaker (MF1-S) was placed into the ear canal. The signals were collected by the electrodes and amplified 20 times with band-pass filters set between 100 Hz and 5 kHz. The signals were input to a real-time processor (RA4PA), processed with BioSig RP software (TDT), and preamplified with a gain of 20 and averaged for 1000 sweeps in each trial. Thresholds were evaluated at 4, 8, 16, 24, and 32 kHz. The stimuli were presented at 90 dB, decreased in 5 dB steps until the threshold was approached, and the ABR wave disappeared for each frequency. The threshold was defined as the lowest intensity at which a visible and repeatable ABR wave was observed in two averaged runs.

### Statistical analysis

GraphPad Prism software (La Jolla, CA, USA) was used for data processing significance analysis. For cell counting, surviving HCs were counted from three or four randomly selected fields from the apical and basal turn of each culture. The average of the cells counted from the randomly selected fields was designated as one sample, and data were collected from three or more cultures for each of the experimental groups. Significant differences in HC number and gene expression were assessed using Student’s *t* test (*p* < 0.05 was considered significant), and ABR thresholds were analyzed by two-way analysis of variance with simplified Bonferroni correction.

## Results

### Pasireotide prevents gentamicin-induced activation of caspase activity in mouse cochlea

**Fig. 1:**
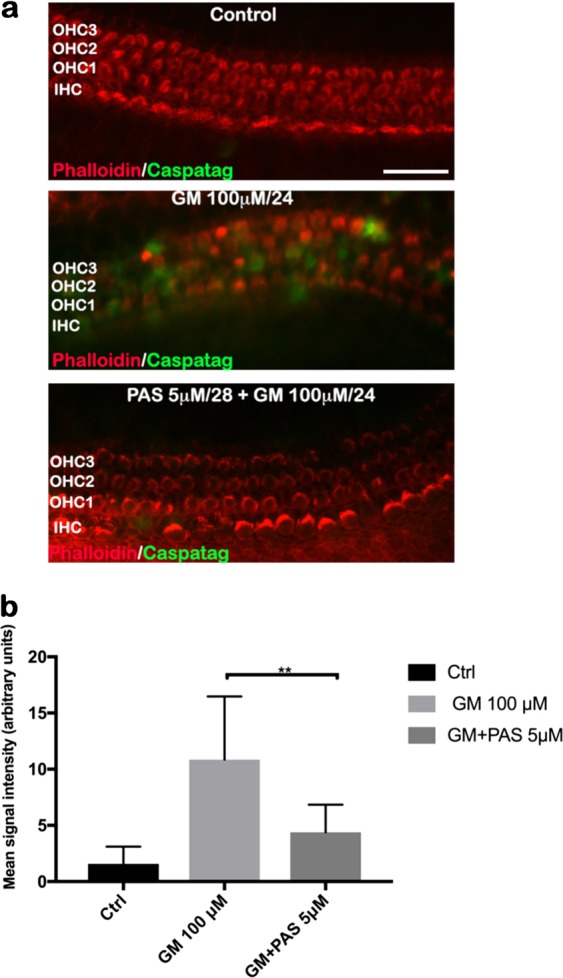
Pasireotide decreased the activation of pro-apoptotic caspases in mouse cochlear HCs after gentamicin injury. **a** Representative fluorescence micrographs of OC basal turns show auditory HCs as detected with rhodamine-phalloidin (red) and activated caspases as detected with Caspatag^50^. Experimental OCs were randomized into three groups (control, CTR; gentamicin, GM; pasireotide, PAS) and treated as described above. Scale bar: 50 μm. **b** Quantification of the fluorescent signal of (**a**). Data were obtained from three independent experiments (*n* = 8; five explants per group from both sexes; **p < 0.0001 by Student’s *t* test). HCs, hair cells; OC, organ of Corti

We have previously demonstrated that the Ca^2+^-sensitive neuropeptide SST and its analog, octreotide, protect HCs from gentamicin-induced apoptosis in vitro^10,12^. In the present study, we report that a next-generation SST analog, pasireotide, prevents gentamicin-induced HC cell death in explants derived from mouse OCs. In the current study, we performed assays to detect active caspases in mouse OC HCs to confirm that HC loss occurred by apoptosis. The OCs were collected from P5 mice from both sexes. Active caspases were assayed with a Caspatag kit in OC explants exposed to 100 µM gentamicin for 24 h with or without 5 µM pasireotide for 28 h. The explants were then fixed and stained with rhodamine-conjugated phalloidin to identify HCs. We found degeneration of IHCs and OHCs caused by gentamicin administration in the basal turn of the cochlea. The intensity of caspase green signal was high. No activated caspases were detected in control OCs (Fig. 1). The intensity of the green signal in control samples was very low. Quantification of the fluorescent signal in panel b showed a significant difference in signal intensity between gentamicin-only and pasireotide-treated OC explants (**p < 0.0001 by Student’s *t* test). Pasireotide prevented caspase activation by gentamicin almost completely in this assay, reflected by the observed signal intensity levels similar to those seen in control untreated OCs.

### Somatostatin antagonists CYN and BIM confirm the high affinity of pasireotides for SSTR2 and SSTR5

**Fig. 2:**
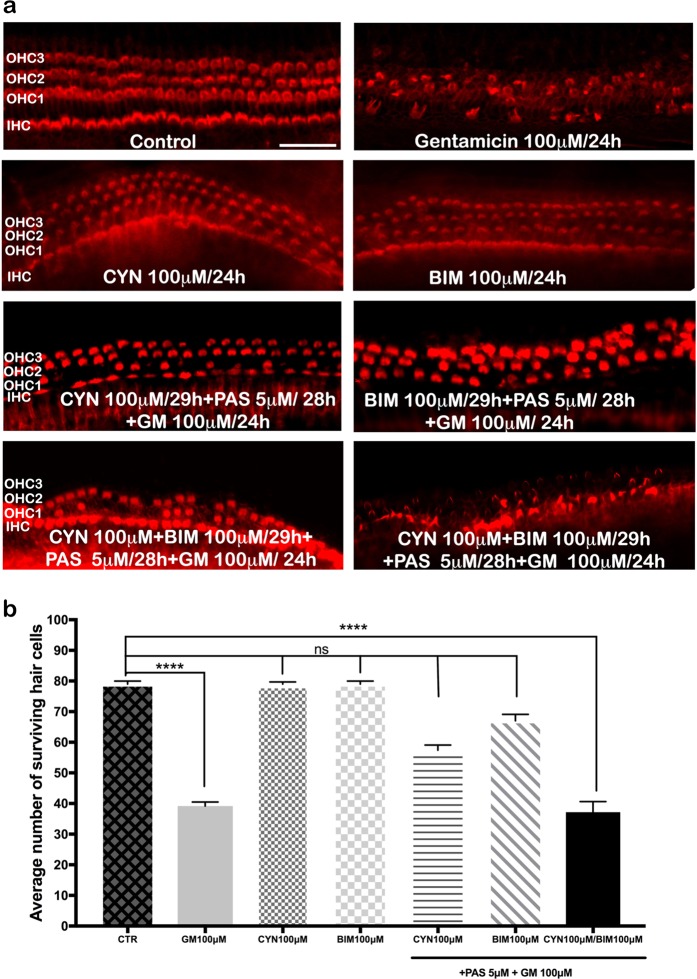
Pasireotide has a high affinity for SST2 and SST5 receptors. **a** Representative fluorescence micrographs of OC basal turns show auditory HCs as detected with Alexa Fluor rhodamine-phalloidin (red). OCs were incubated in the following conditions (top to bottom): medium alone for 48 h; medium for 24 h, then GM (100 μM) for 24 h; CYN-154806 (100 μM) alone for 24 h; BIM (100 μM) alone for 24 h; CYN-154806 (100 μM) with GM (100 μM); BIM (100 μM) with GM (100 μM) and pasireotide (5 µM), GM (50 μM), CYN-154806 (100 μM) and BIM (100 μM) added for the last 28 h. A single treatment with either antagonist does not lead to a high loss of HCs. Scale bar: 50 μm; four OCs per condition; (*n* = 10) from both sexes. **b** Quantitative analysis of HC survival in OC cultures treated with SSTR2 and SSTR5 antagonists. The average number of surviving HCs indicates significant HC loss in cultures treated with pasireotide in combination with both CYN-154806 and BIM compared to control conditions (****p < 0.001 by Student’s *t* test); cells were counted in three independent fields. HCs, hair cells; OC, organ of Corti

Many studies have shown that pasireotide has a high affinity for SSTR2 and SSTR5 using SSTR2- and SSTR5-specific antagonists^15^. Therefore, we questioned whether pasireotide also has an affinity for SSTR2 and SSTR5 in auditory HCs. To address this question, we first blocked these receptors with specific antagonists. In P5 mouse OCs, we studied the effects of the SSTR2 antagonist CYN-154806 and the SSTR5 antagonist BIM on pasireotide activity to investigate the involvement of SST2 and SST5 receptor subtypes in the protective effects of pasireotide (Fig. 2). Co-incubation of pasireotide with CYN-154806 or BIM leads to incomplete HC protection following gentamicin treatment, and a combination of both antagonists and pasireotide reverses paseriotide-induced protection (Fig. 2a). No toxicity was observed for CYN-154806 or BIM alone at a concentration of 100 µM.

Quantification of these results indicated reduced HC loss in the group receiving combined treatment with CYN-154806, BIM-23056, and pasireotide, approximately 50% of that seen when pasireotide was combined with each separately. The average number of surviving HCs indicates significant HC loss in cultures treated with 5 µM pasireotide in combination with both 100 µM CYN-154806 and 100 µM BIM-23056 compared to control conditions (*n* = 10; ****p < 0.001 by Student’s *t* test). The number of HCs in the culture following treatment with CYN-154806 and BIM-23056 antagonists separately compared with the number of HCs in control cultures was higher for 30–40% than in cultures treated with pasireotide combined with both antagonists (Fig. 2b).

### Pasireotide increased the expression of survival genes and activated the PI3K–Akt survival signaling pathway through PTPs

**Fig. 3:**
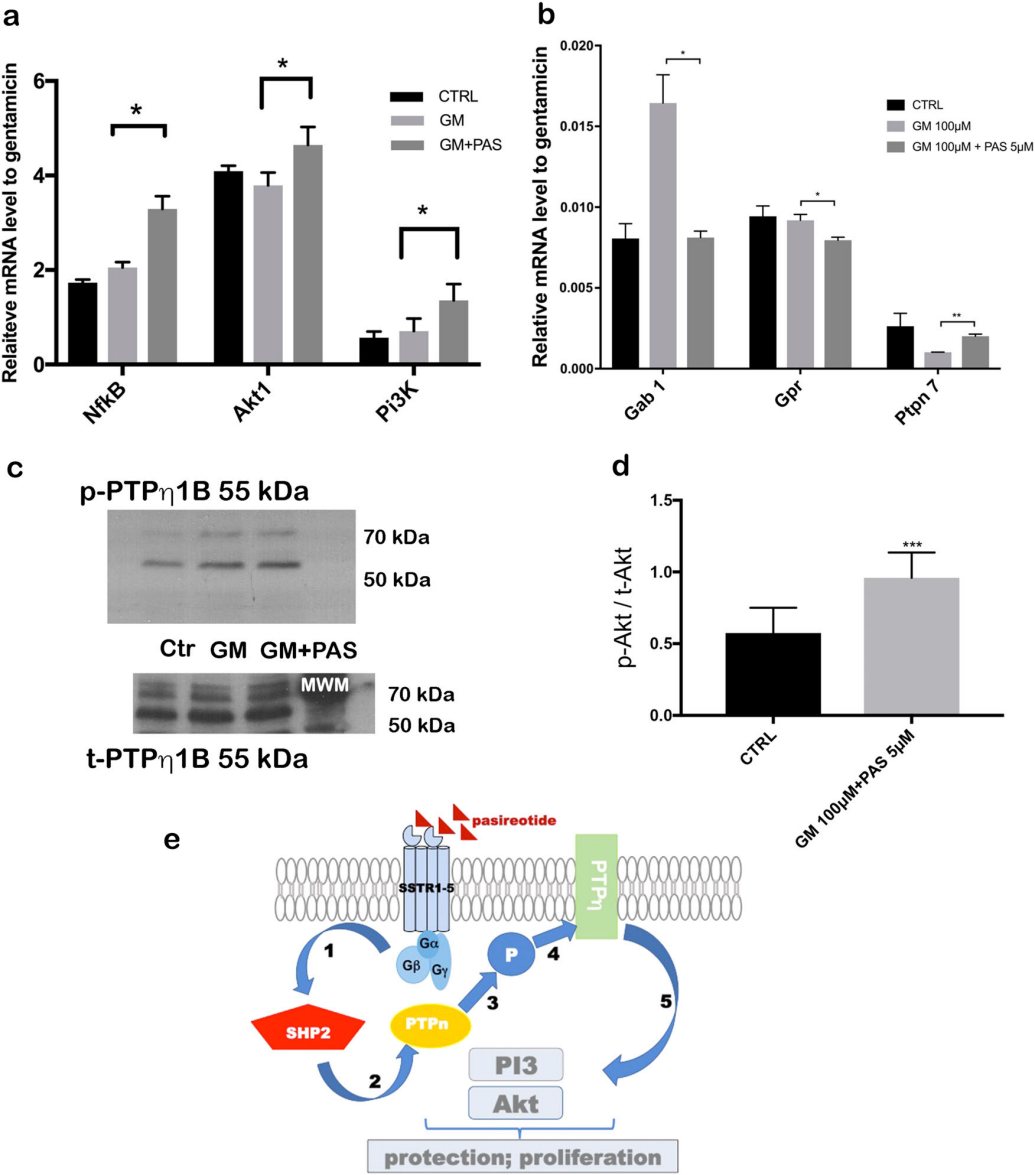
Pasireotide protects HCs from gentamicin toxicity by enhancing survival gene expression. **a** Mouse OCs were treated as described in the Materials and methods section. GM alone had no effect on the relative expression of *NfkB,* *Akt 1*, or *Pi3k,* while pasireotide up-regulated the expression of all the three genes, which correlated with a significant improvement in cell protection. **b** To further elucidate the mechanisms by which pasireotide activated the PI3K–Akt survival signaling pathway, we examined the expression of *Gab1*, G-protein receptor (*Gpr*), and *PTPn7* genes. The relative expression levels of *Gab1*, *Gpr*, and *PTPn7* mRNA in untreated (control), GM-treated, and GM + pasireotide-treated murine OCs are shown. GM strongly induced *Gab1* and *Gpr* mRNA. Thus, these two genes are up-regulated in stress situations, such as ischemia or ototoxicity. The reverse effect was seen with *PTPn7* mRNA with the pasireotide co-treatment. **c** Western blots of lysates prepared from murine OCs that were untreated, treated with GM alone, or treated with GM + pasireotide were performed to detect the activation of Akt. Specific antibodies were used for phosphor-PTPB1 (p-PTPB1) and total PTPB1 (t-PTPB1). **d** Quantification of western blot signals represented by the ratio of p-PTPB1/t-PTPB1 signals for 10 explants per group (*n* = 15), including pups of both sexes (***p < 0.001 by Student’s *t* test). Values represent the mean ± one standard deviation of three biological replicates. **e** Schematic representation of the intracellular signaling pathway modulated by SSTRs. After binding to SSTR2 and SSTR5, pasireotide activated different phosphotyrosine phosphatases (PTPs), such as SHP2 or PTPn (PTPn7). Activated PTPn directly interacted with PTPη inducing phosphorylation and tyrosine activation. PTPη dephosphorylates intracellular effectors, such as the PI3K–Akt pathway involved in the protection of hair cells. GM, gentamicin; OCs, organs of Corti

To determine the mechanism responsible for the protective function of pasireotide, we investigated the expression of survival genes and activation of the PI3K survival signaling pathway. We used qPCR of three biological replicates to investigate the influence of pasireotide on the expression of survival genes, including the *NF-κB* subunit 1, *Akt 1*, the regulatory subunit of Pi3K, as well as the genes involved in the PI3K–Akt survival signaling pathway in OCs from P5 mice of both sexes. Pretreatment with 5 µM pasireotide for 4 h and treatment with 5 µM pasireotide and 100 µM gentamicin for 24 h significantly increased HC survival and activated the survival genes. Survival gene-expression levels were significantly higher in explants that received protective treatment with pasireotide as described above compared with a gentamicin-only group (Fig. 3a). GAPDH was used as a housekeeping gene, and significance was determined (*n* = 15, 10 OC per condition, *p* < 0.05 by Student’s *t* test). Herein, we further demonstrated that the G protein-coupled receptors and *Gab1* (*Grb-2*-associated binder) were expressed in HCs. *Gab1* expression was significantly higher in HCs from explants treated with gentamicin than in those treated with gentamicin + pasireotide. After binding of pasireotide to the SSTRs, different PTPs were activated. The expression level of *PTPn7*, one of the non-transmembrane PTP members, was significantly higher in explants treated with gentamicin + pasireotide compared with that in those treated with gentamicin. This confirms that pasireotide activated the PTPs after binding to the SSTRs (Fig. 3b). Activated PTPs (*PTPn7*) directly interact with *PTPη*, inducing the phosphorylation of tyrosine and activation. Then, *PTPη* dephosphorylates the intracellular effectors, such as the PI3K–Akt pathway involved in HC protection and proliferation. Therefore, we questioned whether PTPη (Β1 subunit) is expressed and could be activated through PTPn7 in auditory HCs. To address this question, we used phosphor-PTPB1 to recognize PTPη phosphorylation and detect the activation of PTPη after the treatment of OC explants with pasireotide. Western blots showed that PTPB1 phosphorylation was increased in pasireotide-treated OC cultures (Fig. 3c). Phospho-PTPB1 levels were significantly increased by pasireotide treatment (*n* = 15, 10 OCs per condition, *p* < 0.05 by Student’s *t* test). The ratio of phospho-PTPB1/total-PTPB1 was used to quantify and normalize phospho-PTPB1 levels (Fig. 3d). An overview of the HC signaling pathway activated by SSTRs, after treatment with pasireotide, is depicted in Fig. 3e.

### Activation of the PI3K–Akt survival signaling pathway after treatment with pasireotide promotes HC survival

**Fig. 4:**
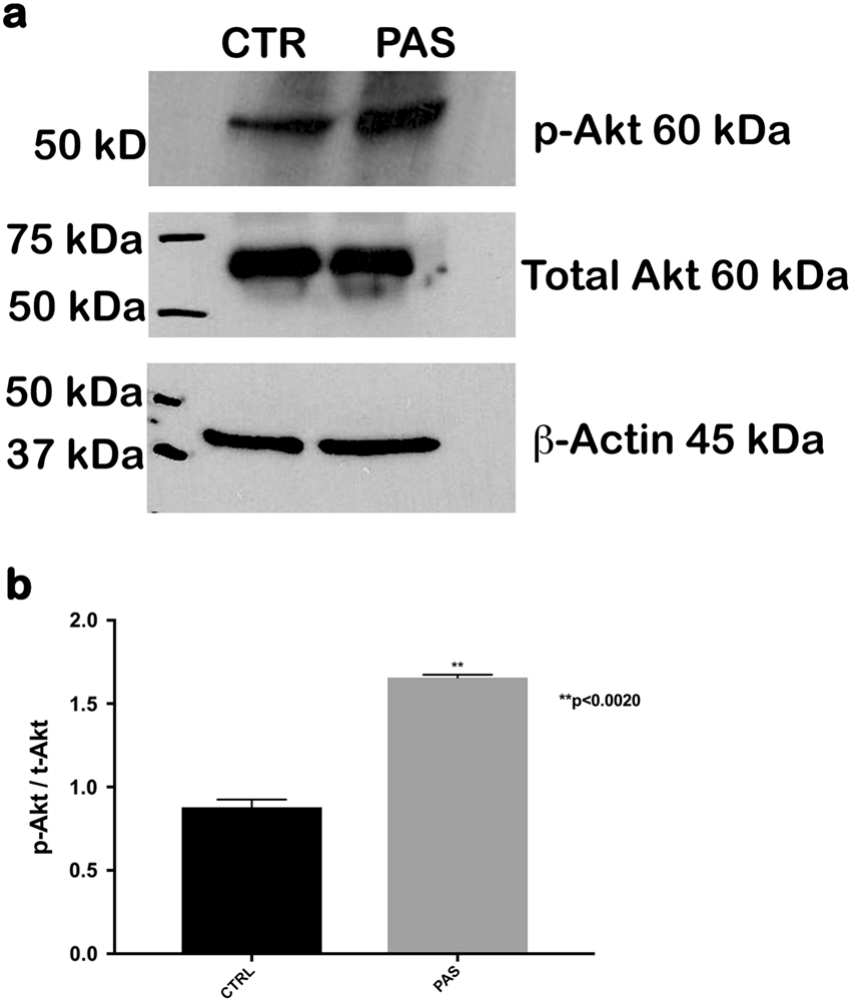
Pasireotide increases Akt phosphorylation in cultivated mouse OCs. **a** OC explants were exposed for 4 h to media containing 5 µM pasireotide followed by 24 h of exposure to media containing 5 µM pasireotide and 100 µM gentamicin. Control OCs were exposed to control media for 24 h. P-Akt levels from OCs treated with pasireotide were standardized against the total Akt. **b** P-Akt levels were significantly increased by pasireotide treatment (**p < 0.05 by Student’s *t* test). Bars show the mean ± one standard deviation of three biological replicates. Ten OCs from five pups in each condition, including both sexes. OCs, organs of Corti

We then explored the potential mechanisms underlying the protective functions of pasireotide through the activation of the PI3K–Akt signaling pathway. To prove pasireotide-induced activation of PI3K signaling, we evaluated changes in AKT phosphorylation in pasireotide-treated and non-treated OC explants using a Akt-phospho-specific antibody, since phospho-AKT levels reflect PI3K signaling activity levels. The P5 mouse OC explants were exposed for 4 h to media containing 5 µM pasireotide followed by 24 h of exposure to media containing 5 µM pasireotide and 100 µM gentamicin. Control OCs were exposed to control media for 24 h. Western blot analysis demonstrated that AKT phosphorylation increased in pasireotide-treated OC cultures (Fig. 4a). P-Akt levels were significantly increased by pasireotide treatment (*n* = 5, 10 OCs per condition, *p* < 0.05 by Student’s *t* test). The ratio of phospho-AKT/total AKT was determined to quantify and normalize phospho-AKT levels (Fig. 4b).

### Specific Akt inhibitor SH-6 reduces the protective effects of pasireotide against gentamicin-induced HC damage in mouse OC culture

**Fig. 5:**
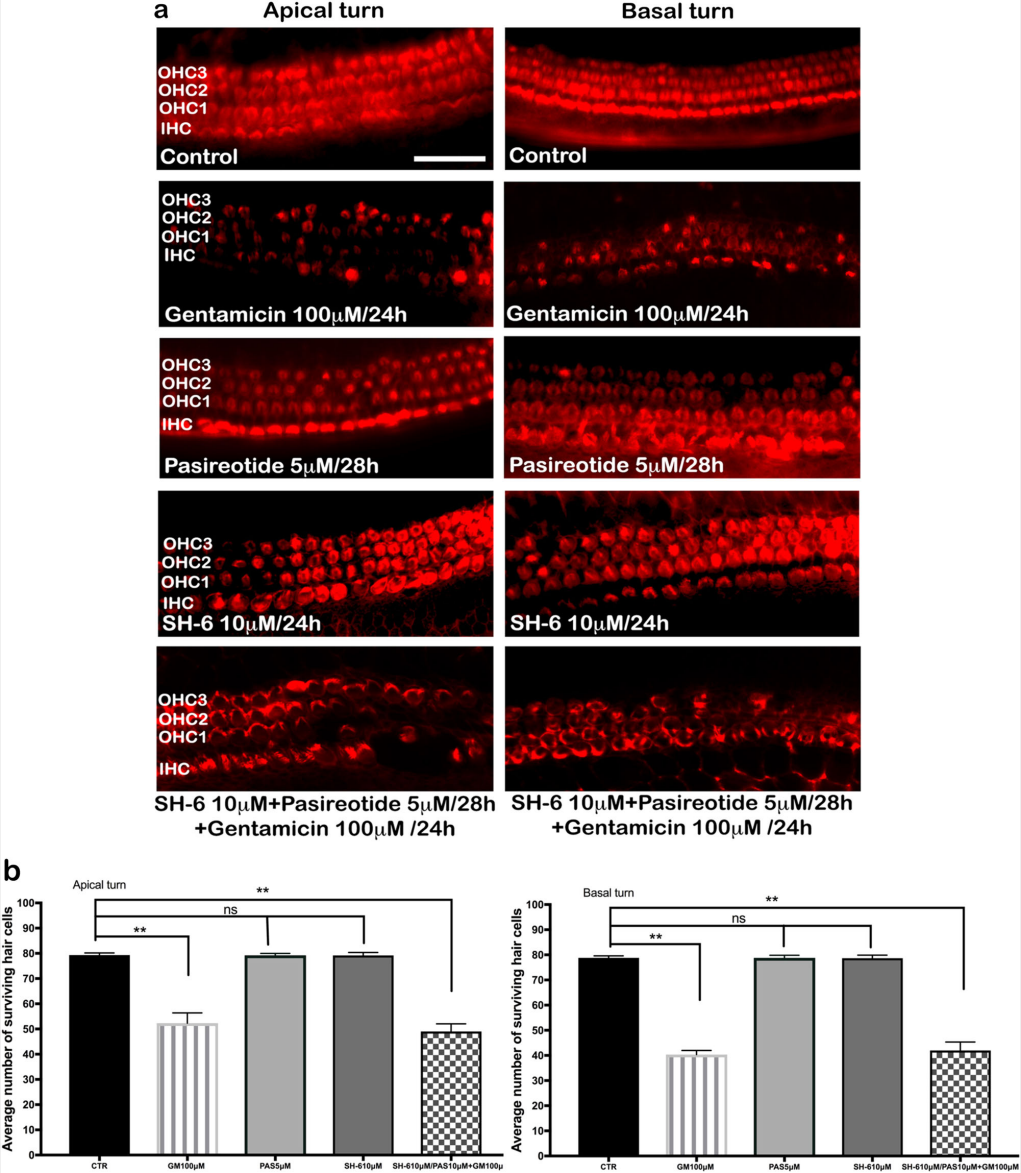
Effects of the Akt inhibitor SH-6 on gentamicin-induced HC damage. **a** Three orderly rows of OHC and a single row of IHC in the apical and basal turn were observed in OCs exposed to 10 mM SH-6, while OCs cultured with 100 µM gentamicin showed significant hair cell loss. The addition of 5 µM pasireotide to 100 µM gentamicin and SH-6 also showed hair cell loss. Scale bar = 50 μm. Four OCs per condition (*n* = 10). **b** Quantitative analysis of HC survival following treatment with the Akt inhibitor SH-6 in apical and **c** basal cochlear turns. No toxic effect was seen when HCs were incubated with SH-6 alone. Data are expressed as the mean number of surviving HCs per 20 inner HCs and 60 outer HCs in three independent fields. **p < 0.001 by Student’s *t* test, compared to the untreated culture (control). HCs, hair cells; IHCs, inner HCs; OC, organ of Corti; OHCs, outer HCs

To confirm that Akt could play an important role in the downstream targeting of PI3K signaling in cochlea, we inhibited its effect with specific SH-6 inhibitor. Treatment of OCs with the Akt inhibitor SH-6 alone did not result in HC damage, and three orderly rows of OHCs and one row of IHCs were seen in the basal and apical turns, respectively (Fig. 5a). However, treatment with 10 µM SH-6 with the addition of 5 µM pasireotide reduced the protective effect of pasireotide. Cell numbers after SH-6 treatment were significantly lower than those in controls (Fig. 5b) (*n* = 10, four OCs per condition, *p* < 0.05 by Student’s *t* test).

### The protective effect of pasireotide against gentamicin requires Akt activation

**Fig. 6:**
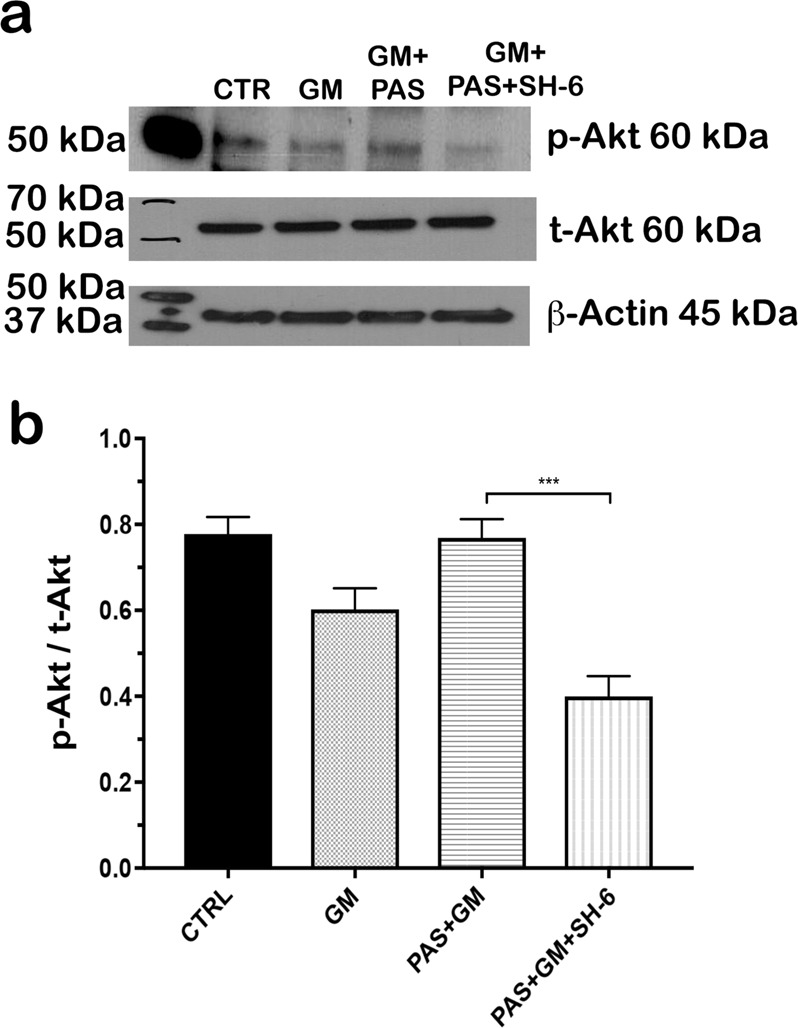
Western blotting shows that the inhibitor SH-6 bound to Akt and blocked the protective action of pasireotide. **a** Western blots of protein extracts prepared from murine OCs after no treatment and treatment with gentamicin alone or in the presence of pasireotide and SH-6 inhibitor. p-Akt signal was very weak in the samples from gentamicin, pasireotide, and SH-6 groups. β-Actin signals were used to demonstrate equal protein loading. **b** Quantification of western blot signals represented by the ratio of p-Akt/t-Akt signals; ten explants per group (*n* = 20), including pups of both sexes (***p < 0.001 by Student’s *t* test). OCs, organs of Corti

To determine whether pasireoitide activated Akt, we performed western blot using the lysates of non-treated, gentamicin-treated, pasireotide-treated, and pasireotide- and SH-6-treated animals. A significantly lower phosphorylated Akt signal was observed in the pasireotide- and SH-6 inhibitor-treated OC explants compared to explants treated with pasireotide alone (Fig. 6a). Western blot signals were quantified by the ratio of p-Akt/t-Akt signals (*n* = 20, 10 OCs per condition, *p* < 0.001 by Student’s *t* test); values are the mean ± SD, normalized to ß-actin (Fig. 6b).

### Efficacy of hair-cell protection after gentamicin injury in vivo as assessed by auditory brainstem response

**Fig. 7:**
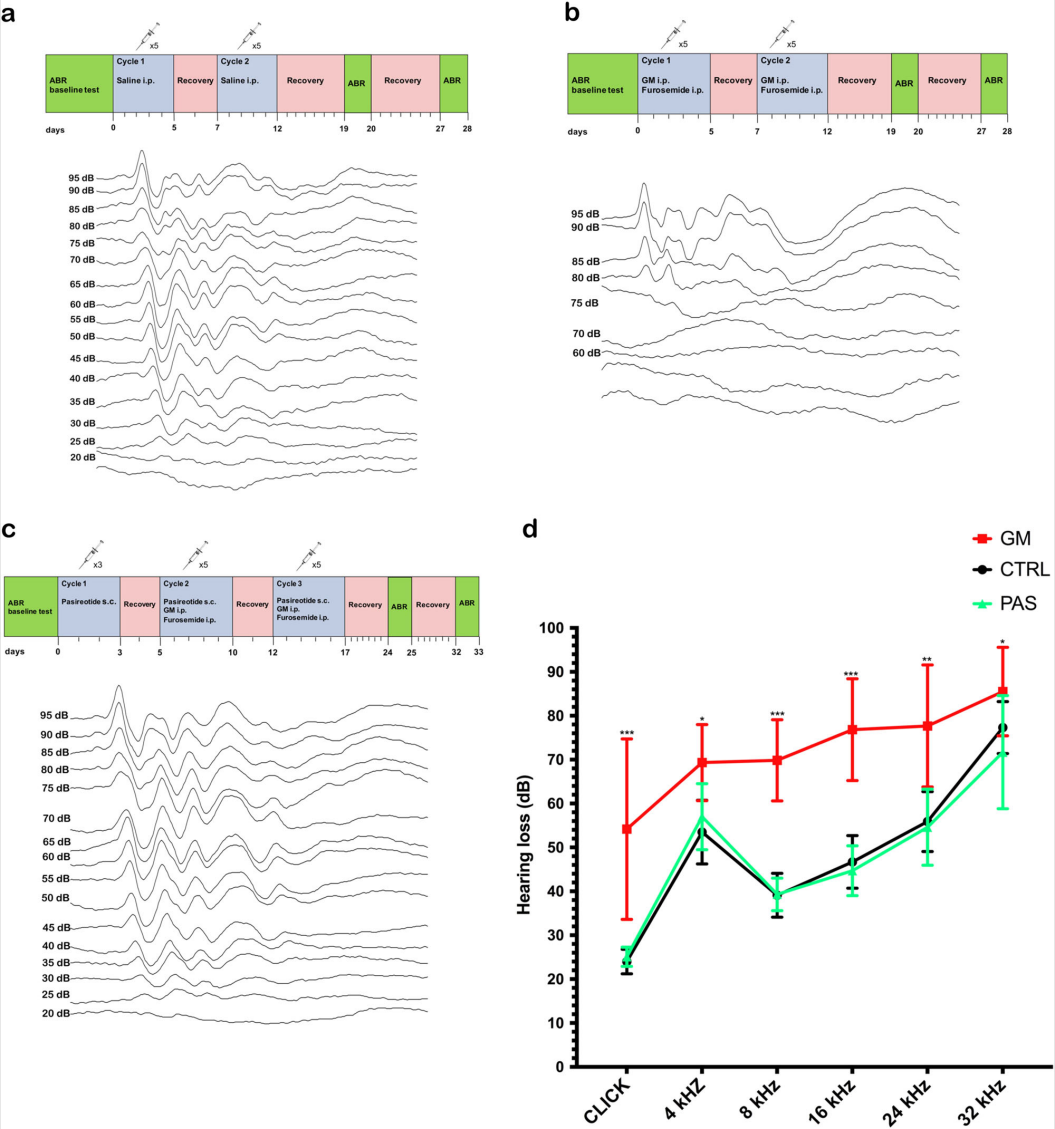
Decreased hearing thresholds 13 days after pasireotide injection. Representative hearing thresholds from **a** control at ~ 25 dB, **b** gentamicin-treated group at ~ 75 dB, and **c** gentamicin + pasireotide-treated group at approximately 25 dB. ABR recordings were performed in *n* = 10–12 animals per group (5–6 males and 5–6 females). The schemas present the drug regimens administered to C57BL/6N mice. The two cycles in the control and gentamicin groups consisted of once-daily i.p. injections of saline or gentamicin for 10 days followed by 2 days recovery after every 5^th^ day. The three-cycle pasireotide regimen consisted of 3 days of once-daily subcutaneous injections of pasireotide followed by 2 days of recovery; 10 days of three i.p. injections of pasireotide, gentamicin, and furosemide followed by 2 days recovery after every 5^th^ day. **d** Summary of ABR measurements. Frequency-dependent hearing thresholds in control (CTR; normal saline), gentamicin (GM), and gentamicin + pasireotide (GM + PAS). Significant differences were found between GM and GM + PAS hearing thresholds at all measured frequencies and clicks (*p < 0.01; **p < 0.01; ***p < 0.001; ****p < 0.001). All statistical analyses of in vivo data were conducted via two-way ANOVA of the parameters between various regimens. ABR, auditory brainstem response; ANOVA, analysis of variance

To extend the studies, we tested the ability of pasireotide to protect hearing function in vivo from gentamicin ototoxicity. We performed auditory-evoked brainstem response recordings after 10 days of injections of saline or gentamicin or 13 days of gentamicin + pasireotide treatment in 2-month-old mice (*n* = 36) to evaluate the protective role of pasireotide on gentamicin-induced hair-cell loss and the subsequent ototoxic host response on hearing function. Mice were tested with broadband click stimuli and pure tones to determine hearing thresholds. Representative click-ABR recordings from control and gentamicin- and pasireotide-treated mice are shown in Fig. 7. No detectable waves were generated below 25 dB for the control and 75 dB for the gentamicin-treated mice (Fig. 7a, b). Mean hearing threshold values were not significantly different from controls following pasireotide treatment (Fig. 7c). A significant difference in threshold shift was found between the gentamicin and gentamicin + pasireotide groups at all measured frequencies and clicks (*p < 0.01; **p < 0.01; ***p < 0.001; ****p < 0.001 by two-way analysis of variance) (Fig. 7d). ABR thresholds in response to click were approximately 75 dB in the gentamicin group compared to 25 dB for untreated controls, corresponding with normal hearing for C57BL/6NCrl mice in the same age range (Fig. 7a, b). All animals received a hearing function test before treatment and 7 and 14 days post-injection, respectively. No significant differences between the groups were found after 7 days ABR. No differences were seen between male and female animals in any group. This finding indicates the great potential of pasireotide as a therapeutic drug.

## Discussion

HC loss and protection are central problems for hearing studies. We report that the novel peptide drug pasireotide protects HCs against gentamicin ototoxicity by activating the PI3K–Akt pathway and reducing caspase activity both in vitro and in vivo. In our previous study, we demonstrated that gentamicin induced apoptotic death of HCs, by the production of reactive oxygen species, lipid peroxidation, and caspase activation^26^. HC counts and caspase staining of OC explants exposed to the gentamicin reveal that many of the ototoxin-damaged HCs show typical characteristics of apoptosis (Fig. 1), in agreement with many studies reporting apoptosis of aminoglycoside-damaged HCs^27,26^. Aminoglycosides initially stimulate an influx of calcium ions and the associated rapid rise in intracellular HC Ca^2+^ concentration^28^. The Ca^2+^-sensitive neuropeptide SST and its five receptors are expressed in the mammalian inner ear, specifically in the OHCs and IHCs of OCs, in supporting cells, and in spiral ganglion cells^29,30^. Pasireotide is a new-generation SST agonist that binds to SSTRs 1–3 and 5 with a high affinity and has an improved, longer half-life^15^. This agent was found to be extremely effective in protecting HCs in an in vitro model of mouse OC exposure to gentamicin^11^, extending our previous reports that native SST, as well as the less-active analog octreotide, protects HCs from gentamicin-induced hair-cell death in vitro^10,12^. The binding affinity of pasireotide is 30- to 100-fold greater than either octreotide or lanreotide^31^. The significant cytoprotective effects exerted by pasireotide reported here confirm parallel observations in models of retinal protection, in which pasireotide was more efficacious than either octreotide or lanreotide^31^.

The neuroprotective effects of pasireotide in the cochlea are mainly due to the involvement of SSTR2 and SSTR5 receptors. We studied the effect of SSTR2 and SSTR5 antagonists to examine the involvement of both receptor subtypes in the protective effects of pasireotide. As shown in Fig. 2, incubation with a single antagonist partially blocked pasireotide-induced protection, while the combination of both antagonists reversed the protection. A similar effect was shown in a study of the neuroprotective effects of pasireotide in two models of retinal ischemia^32^.

Our previous studies have demonstrated that the PI3K–Akt pathway is involved in the protection and survival of auditory HCs^22,33^. Activated Akt has been detected in several inner ear structures, indicating that Akt plays a role in inner ear physiology^34^. In recent years, several cell-survival pathways have been characterized, including the PI3K pathway, which leads to Akt activation^35^. Akt plays a central role in promoting the survival of a wide range of cell types through various mechanisms^36^ and acts as an anti-apoptotic agent by affecting many downstream effectors of programmed cell death, such as BAD, FKHR, and caspase-3^37^. We determined that the PI3K–Akt pathway is involved in pasireotide-induced protection of HCs from gentamicin toxicity in OC explants (Fig. 3), and SSTRs are known to interact with heterotrimeric G proteins following activation by SST and its analog^13^. Several key enzymes are involved, including PTPs and adenylyl cyclase, as well as pathways including mitogen-activated protein kinase (MAPK) and phosphoinositol-3-kinase (PI3K)/Akt, which are modulated along with the reduction of the Ca^2+^ influx through voltage-sensitive channels^38^. To further elucidate the mechanisms by which pasireotide activates the PI3K–Akt pathway, we examined the expression of *Gab1*, G-protein receptors, and *PTPn7* genes, which may help protect HCs from gentamicin toxicity. *Gab1* is known as a key player in insulin signal transduction as a substrate of epidermal growth factor and insulin receptors^39,40^. Many studies have demonstrated that *Gab1* is up-regulated in the injured cells within the ischemic zone, becomes highly phosphorylated, and subsequently activates Akt and MAPK depending on the activities of the receptor-type and non-receptor-type tyrosine kinases^41,42^. The *PTPn* mRNA was also up-regulated in the OC explants treated with gentamicin + pasireotide (Fig. 3b). After binding of SST or its analogs to the SSTRs, different PTPs were activated^43^. The expression level of *PTPn7*, one of the non-transmembrane PTP members, was significantly higher in explants treated with gentamicin + pasireotide compared with those treated with gentamicin. This confirms that pasireotide activated the PTPs after binding to the SSTRs (Fig. 3b). Activated PTPs (*PTPn7*) directly interact with PTPη, inducing the phosphorylation of tyrosine and activation. Then, PTPη dephosphorylates intracellular effectors, such as the PI3K–Akt pathway involved in HC protection and proliferation. Finally, the western blot results from OC lysates treated with gentamicin + pasireotide clearly showed phosphorylated PTPB1 antibody signals and confirmed the activation of the PI3k–Akt signaling pathway (Fig. 3e). The present data strongly support the specific activation of Akt in OCs treated with pasireotide in vitro (Fig. 4), and pasireotide increases Akt activation. The protective effect of pasireotide on gentamicin-induced HC damage was reversed when the Akt inhibitor SH-6 was added to the OC culture.

A major downstream effector of Akt is nuclear factor-kappa B (NF-κB), which can link Akt signaling to cell nucleus. NF-κB also appears to act in feedback on Akt activation, as its inhibition leads to a reduced p-Akt/Akt ratio^44^. In our previous study, we have found a constitutively active form of NF-κB in the OC of 5-day-old rats and that the selective inhibition of NF-κB in vitro caused massive degeneration of HCs within 24 h of inhibitor application^45^. Recent studies indicate the role of NF-κB in mature cochlea. A study conducted with adult mice has demonstrated that the redox state of the cochlea stimulates NF-κB activation^46^. In another study, adult mice lacking the p50 NF-κB subunit suffered from increased noise-induced hearing loss compared to their wild-type littermates^47^. Thus, NF-κB seems to play an important role in adult mammalian cochlea. In this study, we reported that pasireotide induced high expression of *NF-κB* contributing to the protection of HCs after gentamicin exposure. These data suggest the important role of NF-κB in mediating the survival of immature auditory HCs.

In vivo data indicate significant differences in auditory thresholds at all measured frequencies (4, 8, 16, 24, and 32 kHz) and clicks between the gentamicin-treated group and the gentamicin + pasireotide-treated group. The protective effect was not evident on the 7th day, likely because HC loss was not significant at this time point (data not shown). It is generally believed that apical HCs are more resistant to damage than those in the base^3,48^. Previous studies have shown that HC damage is higher 2 weeks post gentamicin treatment^49^. However, HC loss was more evident and the protective effects of pasireotide became significant on the 14th day following gentamicin administration based on the difference in the numbers of surviving HCs and ABR measurments.

The otoprotective effects of pasireotide were demonstrated by systemic delivery in mice. The biggest hearing threshold shifts were seen at high frequencies, in agreement with the observed hearing loss in gentamicin-treated mice (Fig. 7). No significant threshold differences were found between the untreated control and pasireotide groups, while both the control and pasireotide groups showed significant threshold differences at 8, 16, and 24 kHz compared to the gentamicin group. Pretreatment and treatment with pasireotide prevented gentamicin-induced HC toxicity.

The results from all these preclinical in vitro and in vivo studies underscore the necessity of further investigation of pasireotide in clinical studies.

## References

[1] Gillespie PG, Walker RG (2001). Molecular basis of mechanosensory transduction. Nature.

[2] Ruben RJ (1967). Development of the inner ear of the mouse: a radioautographic study of terminal mitoses. Acta Otolaryngol..

[3] Schacht J, Talaska AE, Rybak LP (2012). Cisplatin and aminoglycoside antibiotics: hearing loss and its prevention. Anat. Rec. (Hoboken)..

[4] Wang Q, Steyger PS (2009). Trafficking of systemic fluorescent gentamicin into the cochlea and hair cells. J. Assoc. Res. Otolaryngol..

[5] Imamura S, Adams JC (2003). Distribution of gentamicin in the guinea pig inner ear after local or systemic application. J. Assoc. Res. Otolaryngol..

[6] Alharazneh A (2011). Functional hair cell mechanotransducer channels are required for aminoglycoside ototoxicity. PLoS One.

[7] Tran Ba Huy P, Bernard P, Schacht J (1986). Kinetics of gentamicin uptake and release in the rat. Comparison of inner ear tissues and fluids with other organs. J. Clin. Invest..

[8] Forge A (1985). Outer hair cell loss and supporting cell expansion following chronic gentamicin treatment. Hear. Res..

[9] Law SF, Woulfe D, Reisine T (1995). Somatostatin receptor activation of cellular effector systems. Cell. Signal..

[10] Caelers A, Monge A, Brand Y, Bodmer D (2009). Somatostatin and gentamicin-induced auditory hair cell loss. Laryngoscope.

[11] Bodmer D, Perkovic A, Sekulic-Jablanovic M, Wright MB, Petkovic V (2016). Pasireotide prevents nuclear factor of activated T cells nuclear translocation and acts as a protective agent in aminoglycoside-induced auditory hair cell loss. J. Neurochem..

[12] Brand Y (2014). Role of somatostatin receptor-2 in gentamicin-induced auditory hair cell loss in the Mammalian inner ear. PLoS One.

[13] Patel YC (1999). Somatostatin and its receptor family. Front. Neuroendocrinol..

[14] Moller, L. N., Stidsen, C. E., Hartmann, B. & Holst, J. J. Somatostatin receptors. *Biochim. Biophys. Acta*. 22; **1616**, 1–84 (2003). 10.1016/s0005-2736(03)00235-9 14507421

[15] Schmid HA (2008). Pasireotide (SOM230): development, mechanism of action and potential applications. Mol. Cell. Endocrinol..

[16] Ferjoux G (2000). Signal transduction of somatostatin receptors negatively controlling cell proliferation. J. Physiol. Paris.

[17] Florio T (2008). Somatostatin/somatostatin receptor signalling: phosphotyrosine phosphatases. Mol. Cell. Endocrinol..

[18] Aburto MR, Magarinos M, Leon Y, Varela-Nieto I, Sanchez-Calderon H (2012). AKT signaling mediates IGF-I survival actions on otic neural progenitors. PLoS One.

[19] Barbieri F (2013). Peptide receptor targeting in cancer: the somatostatin paradigm. Int. J. Pept..

[20] Bodmer D, Brors D, Pak K, Gloddek B, Ryan A (2002). Rescue of auditory hair cells from aminoglycoside toxicity by Clostridium difficile toxin B, an inhibitor of the small GTPases Rho/Rac/Cdc42. Hear. Res..

[21] Brand Y (2011). Simvastatin protects auditory hair cells from gentamicin-induced toxicity and activates Akt signaling in vitro. BMC. Neurosci..

[22] Brand Y (2015). All Akt isoforms (Akt1, Akt2, Akt3) are involved in normal hearing, but only Akt2 and Akt3 are involved in auditory hair cell survival in the mammalian inner ear. PLoS One.

[23] Liang J, Slingerland JM (2003). Multiple roles of the PI3K/PKB (Akt) pathway in cell cycle progression. Cell Cycle.

[24] Manning BD, Cantley LC (2007). AKT/PKB signaling: navigating downstream. Cell.

[25] Sobkowicz HM, Loftus JM, Slapnick SM (1993). Tissue culture of the organ of Corti. Acta Otolaryngol. Suppl..

[26] Sekulic-Jablanovic M (2017). Effects of peroxisome proliferator activated receptors (PPAR)-gamma and -alpha agonists on cochlear protection from oxidative stress. PLoS One.

[27] Forge A, Li L (2000). Apoptotic death of hair cells in mammalian vestibular sensory epithelia. Hear. Res..

[28] Dulon D, Zajic G, Aran JM, Schacht J (1989). Aminoglycoside antibiotics impair calcium entry but not viability and motility in isolated cochlear outer hair cells. J. Neurosci. Res..

[29] Radojevic V (2011). The somatostatinergic system in the mammalian cochlea. BMC. Neurosci..

[30] Bodmer, D., Brand, Y. & Radojevic, V. Somatostatin receptor types 1 and 2 in the developing mammalian cochlea. *Dev Neurosci*. 10.1159/000341291 (2012). 10.1159/000341291 22986312

[31] Schmid HA, Schoeffter P (2004). Functional activity of the multiligand analog SOM230 at human recombinant somatostatin receptor subtypes supports its usefulness in neuroendocrine tumors. Neuroendocrinology.

[32] Kiagiadaki F, Thermos K (2008). Effect of intravitreal administration of somatostatin and sst2 analogs on AMPA-induced neurotoxicity in rat retina. Invest. Ophthalmol. Vis. Sci..

[33] Bodmer D (2008). Protection, regeneration and replacement of hair cells in the cochlea: implications for the future treatment of sensorineural hearing loss. Swiss Med. Wkly.

[34] Hess A, Labbe D, Watanabe K, Bloch W, Michel O (2006). Evidence for an Akt-kinase/NO/cGMP pathway in the cochlea of guinea pigs. Eur. Arch. Otorhinolaryngol..

[35] Fruman DA, Meyers RE, Cantley LC (1998). Phosphoinositide kinases. Annu. Rev. Biochem..

[36] Kennedy SG (1997). The PI 3-kinase/Akt signaling pathway delivers an anti-apoptotic signal. Genes Dev..

[37] Orike N (2001). Role of PI 3-kinase, Akt and Bcl-2-related proteins in sustaining the survival of neurotrophic factor-independent adult sympathetic neurons. J. Cell. Biol..

[38] Florio T (2008). Molecular mechanisms of the antiproliferative activity of somatostatin receptors (SSTRs) in neuroendocrine tumors. Front. Biosci..

[39] Sun L (2014). Grb2-associated binder 1 is essential for cardioprotection against ischemia/reperfusion injury. Basic. Res. Cardiol..

[40] Rocchi S (1998). Determination of Gab1 (Grb2-associated binder-1) interaction with insulin receptor-signaling molecules. Mol. Endocrinol..

[41] Holgado-Madruga M, Wong AJ (2003). Gab1 is an integrator of cell death versus cell survival signals in oxidative stress. Mol. Cell. Biol..

[42] Chan PC, Sudhakar JN, Lai CC, Chen HC (2010). Differential phosphorylation of the docking protein Gab1 by c-Src and the hepatocyte growth factor receptor regulates different aspects of cell functions. Oncogene.

[43] Tiganis T, Bennett AM (2007). Protein tyrosine phosphatase function: the substrate perspective. Biochem. J..

[44] Caelers A, Radojevic V, Traenkle J, Brand Y, Bodmer D (2010). Stress and Survival Pathways in the Mammalian Cochlea. Audiol. Neurootol..

[45] Nagy I, Monge A, Albinger-Hegyi A, Schmid S, Bodmer D (2005). NF-kappaB is required for survival of immature auditory hair cells in vitro. J. Assoc. Res. Otolaryngol..

[46] Jiang H, Sha SH, Schacht J (2005). NF-kappaB pathway protects cochlear hair cells from aminoglycoside-induced ototoxicity. J. Neurosci. Res..

[47] Lang H (2006). Nuclear factor kappaB deficiency is associated with auditory nerve degeneration and increased noise-induced hearing loss. J. Neurosci..

[48] Lee JH (2013). Different uptake of gentamicin through TRPV1 and TRPV4 channels determines cochlear hair cell vulnerability. Exp. Mol. Med..

[49] McFadden SL, Ding D, Jiang H, Salvi RJ (2004). Time course of efferent fiber and spiral ganglion cell degeneration following complete hair cell loss in the chinchilla. Brain Res..

[50] Beaudet A, Greenspun D, Raelson J, Tannenbaum GS (1995). Patterns of expression of SSTR1 and SSTR2 somatostatin receptor subtypes in the hypothalamus of the adult rat: relationship to neuroendocrine function. Neuroscience.

